# Differential Capability of Clinically Employed Dermal Regeneration Scaffolds to Support Vascularization for Tissue Bioengineering

**DOI:** 10.3390/biomedicines9101458

**Published:** 2021-10-13

**Authors:** Chiara Agostinis, Mariagiulia Spazzapan, Roman Vuerich, Andrea Balduit, Chiara Stocco, Alessandro Mangogna, Giuseppe Ricci, Giovanni Papa, Serena Zacchigna, Roberta Bulla

**Affiliations:** 1 Institute for Maternal and Child Health, Istituto di Ricovero e Cura a Carattere Scientifico (IRCCS) “Burlo Garofolo”, 34137 Trieste, Italy; chiara.agostinis@burlo.trieste.it (C.A.); alessandro.mangogna@burlo.trieste.it (A.M.); giuseppe.ricci@burlo.trieste.it (G.R.); 2 Department of Life Sciences, University of Trieste, 34127 Trieste, Italy; mariagiulia.spazzapan@studenti.units.it (M.S.); roman.vuerich@icgeb.org (R.V.); rbulla@units.it (R.B.); 3 International Centre for Genetic Engineering and Biotechnology, Cardiovascular Biology Laboratory, 34149 Trieste, Italy; serena.zacchigna@icgeb.org; 4 Department of Medical, Surgical and Health Sciences, University of Trieste, 34100 Trieste, Italy; chiarastoccomd@gmail.com (C.S.); giovanni.papa@asugi.sanita.fvg.it (G.P.)

**Keywords:** endothelial cells, acellular dermal matrices (ADMs), chronic wounds, wound healing, angiogenesis

## Abstract

The loss of skin integrity has always represented a major challenge for clinicians dealing with dermal defects, such as ulcers (diabetic, vascular and chronic), postoncologic resections (i.e., radical vulvectomy) or dermatologic disorders. The introduction in recent decades of acellular dermal matrices (ADMs) supporting the repair and restoration of skin functionality represented a significant step toward achieving clean wound repair before performing skin grafts. Hard-to-heal ulcers generally depend on local ischemia and nonadequate vascularization. In this context, one possible innovative approach could be the prevascularization of matrices with vessel-forming cells (inosculation). This paper presents a comparative analysis of the most widely used dermal templates, i.e., Integra^®^ Bilayer Matrix Wound Dressing, PELNAC^®^, PriMatrix^®^ Dermal Repair Scaffold, Endoform^®^ Natural Dermal Template, and Myriad Matrix^®^, testing their ability to be colonized by human adult dermal microvascular endothelial cells (ADMECs) and to induce and support angiogenesis in vitro and in vivo. By in vitro studies, we demonstrated that Integra^®^ and PELNAC^®^ possess superior pro-adhesive and pro-angiogenetic properties. Animal models allowed us to demonstrate the ability of preseeded ADMECs on Integra^®^ to promote the engraftment, integration and vascularization of ADMs at the site of application.

## 1. Introduction

The loss of skin integrity has always represented a major challenge for clinicians, especially when dealing with fragile patients. Burns, ulcers (diabetic, vascular and chronic), postoncologic resections (i.e., radical vulvectomy with inguinofemoral lymphadenectomy followed by chemoradiotherapy) and dermatologic disorders (i.e., epidermolysis bullosa) are among the most common causes of skin integrity defects [1,2,3]; these conditions represent major disabilities for patients, and have the potential to be fatal in severe cases.

The treatment of skin lesions can be either conservative or surgical, depending on the size and type of wound, as well as on patient characteristics and comorbidities. Generally, when dealing with vulnerable patients, the decision to perform surgery rather than applying conservative treatment is not always straightforward, since patient overall health status has a preponderant influence on the healing process. In fact, several local and systemic factors (immunosuppressive status, malnutrition, hematologic disorders), pathologies and drugs can interfere with the curative process [4]. For instance, pregnancy causes an immunosuppressive state which may contribute to the development of severe necrotic soft tissue infections [5,6]. Metabolic diseases including diabetes can lead to macro/microvascular impairment with low to no healing potential and increased rates of postsurgical infection [7,8]. Diabetes mellitus was observed in 34.7% of patients admitted to gynecology and obstetrics services for necrotizing fasciitis [9]. Alterations in coagulation [10] and malnutrition are also among the factors affecting the wound healing process [10,11,12].

In addition, due to our ageing society and associated chronic diseases, the incidence of ulcers is steadily increasing, causing significant impairment of patient quality of life and having a major economic impact on the health care system [13,14,15].

The introduction in recent decades of acellular dermal matrices (ADMs) as skin substitutes represented major progress in providing coverage and support to repairing and restoring skin functionality [16]. Since AMDs are a substitute for the dermis, subsequent epidermal coverage is usually needed, i.e., through a skin graft. The urgent need for new skin substitutes is a result of the fact that highly invasive procedures, such as traditional autologous skin grafts, are not always possible or indicated [17], as in cases of extensive burns [15]. Moreover, a skin graft can only be successful in the presence of a clean and well vascularized wound bed. At present, several ADMs are commercially available, making it easier to restore and cover clean wounds before performing skin grafts.

The use of commercial biocompatible scaffolds or dermal substitutes in regenerative medicine has been widely investigated, and several research groups are currently developing new biomaterials. One of the main applications of these matrices is the treatment of chronic wounds, ulcers or burns, in an attempt to mimic the mechanical properties of physiological skin and avoid immune rejections or toxicity [18].

A pivotal factor for the wound healing process is graft revascularization. In fact, hard-to-heal ulcers generally depend on local ischemia, due to the formation of an impaired capillary network, with consequent low oxygenation levels and poor nutrient supply [19]. If a macro-ischemia is present, surgery and other medical approaches can be performed, even though current surgical and nonsurgical interventions are not sufficient to overcome the condition in cases of microangiopathy. In the field of tissue engineering, considerable efforts have been made to develop a more effective and feasible treatment. One possible innovative approach is the prevascularization of matrices with vessel-forming cells (inosculation). After preseeding onto the matrix, cells should form vessels; ultimately, these fully-blood perfused constructs can be implanted back into the patient [20,21]. Despite the technical difficulties of the vascular transplantation procedure, it would grant immediate blood supply to the transplant, thus minimizing ischemic injury.

The angiogenetic process plays a fundamental role in physiological events, such as development, wound repair and reproduction, but also in several pathological statuses, such as tumor progression. Usually, endothelial cells (ECs) remain in a quiescent state, but during angiogenesis, they can rapidly proliferate, migrate and organize themselves into tubular structures. Vascular endothelial growth factor-A (VEGF-A) plays a major role in the process via its specific receptor VEGFR-2, also known as Flk-1 or KDR, with contributions from angiopoietin-1 (ANGPT-1), integrins and several chemokines [22,23,24]. The ANGPT-1-Tie (TEK) system has been shown to play a role in controlling the quiescence of adult vasculature, and is involved in the later steps of the signaling cascade that leads to vessel maturation. Furthermore, in nonresting ECs, this pathway may promote 3D capillary organization, proliferation and migration, whereas the system seems to inhibit EC permeability and enhance an anti-inflammatory phenotype [25].

This report presents a comparative analysis of the most widely used dermal templates, i.e., Integra^®^ Bilayer Matrix Wound Dressing, PELNAC^®^, PriMatrix^®^ Dermal Repair Scaffold, Endoform^®^ Natural Dermal Template, and Myriad Matrix^®^ (Table 1), testing their ability to be colonized by human adult dermal microvascular ECs (ADMECs) and to induce and support angiogenesis, both in vitro and in vivo, using a mouse model of full-thickness skin wounds.

## 2. Materials and Methods

### 2.1. Reagents and Antibodies

The following antibodies were used: rabbit mAb antiplatelet EC adhesion molecule (PECAM)-1/CD31, mouse mAb anti-CD146, mouse mAb antivimentin, and rabbit antihuman Ki-67 purchased from Sigma Aldrich (St. Louis, MO, USA); mouse mAb anti-von Willebrand Factor (vWF), goat antimouse fluorescein isothiocyanate (FITC)-conjugated F(ab)’, rabbit mAb anticytokeratin (CK)8/18, mouse mAb anti-Ki-67, and mouse mAb antihuman CD31 from Dako (Milan, Italy); mouse mAb antipodoplanin was bought from Abcam (Cambridge, UK); mouse mAb antivascular endothelial growth factor receptor (VEGFR)-3 from Merk (Darmstadt, Germany); mouse mAb antivascular endothelial (VE)-cadherin obtained through the courtesy of E. Dejana (Mario Negri Institute, Milan, Italy); mouse mAb anti-CD105 FITC-conjugated from ImmunoTools (Friesoythe; Germany); rabbit antihuman vimentin was bought from Santa Cruz Biotechnologies (Dallas, Texas, USA); FITC-conjugated goat antirabbit and cyanin 3 (Cy3)-conjugated goat antirabbit were purchased from Jackson ImmunoResearch (Milan, Italy); goat mAb antimouse PECAM-1/CD31 from R&D Systems (Minneapolis, MN, USA), and antimouse IRdye^®^800CW, antirabbit IRdye^®^680RD, antirabbit IRdye^®^800CW, and antimouse IRdye^®^680RD were bought from LI-COR Biosciences (Lincoln, NE, USA); mouse monoclonal antibody antihuman CD31 (#M0823 Dako), goat monoclonal antibody antimouse CD31 (Goat monoclonal antibody antimouse CD31/PECAM-1 (#AF3628 R&D Systems). All chemicals were purchased by Sigma Aldrich.

### 2.2. Cell Isolation and Culture

Human umbilical vein ECs (HUVECs) were isolated following the protocol described by Jeffe et al. [26]. Pregnant women were enrolled at the Institute for Maternal and Child Health, IRCCS Burlo Garofolo, Trieste, Italy. The study was reviewed and approved by the Regional Ethical Committee of FVG (CEUR), Udine, Italy (Prot. 0010144/P/GEN/ARCS 2019). Informed consent for participation in the study was obtained from all women. Human ADMECs were harvested from skin biopsies of patients undergoing reductive plastic surgery, following the protocol described by Kraling et al. [27]. Patients were enrolled at the Cattinara Hospital of Trieste (Plastic Surgery Unit); informed consent for participation in the study was obtained from all women. Cells were seeded onto a fibronectin-gelatin coated flask and maintained in culture with human endothelial serum free medium (HESFM, Gibco, Carlsbad, CA, USA) supplemented with 20 ng/mL basic fibroblast growth factor, 10 ng/mL epidermal growth factor (Immunological Sciences), 1% penicillin-streptomycin (Sigma Aldrich), 10% *v*/*v* fetal bovine serum (FBS, Life Technologies), 10% *v/v* human serum (Sigma Aldrich) and 1:100 hydrocortisone (Sigma Aldrich). ECs were positively selected with Dynabeads^®^ CD31-conjugated magnetic beads (Invitrogen, Thermo Fisher Scientific-11155D). ADMECs were maintained at 37 °C in a humidified atmosphere in a 5% *v*/*v* CO_2_ incubator.

### 2.3. Immunofluorescence

Cells were seeded onto round glass coverslips of 10 mm diameter previously coated with fibronectin-gelatin (2 μg/cm^2^). Confluent cells were fixed with 3% paraformaldehyde (PFA) for 15 min in the dark. In order to perform quenching, blocking and permeabilization, cells were then incubated with a solution of 1% bovine serum albumin (BSA), 0.1 % Triton X-100 and 50 mM glycine in Dulbecco’s Phosphate Saline Buffer (dPBS, Sigma Aldrich) for 30 min at room temperature (RT). Primary antibodies diluted in dPBS-2% BSA and 0.7 mM CaCl_2_ and 0.7 mM MgCl_2_^+^ were added for 1 h at RT; secondary antibodies were incubated for 30 min at RT. Nuclei were stained with DAPI. Glass slides were mounted with a fluorescence mounting medium (Dako), and images were acquired with a Leica DM3000 microscope (Leica, Wetzlar, Germany) using a Leica DFC320 digital camera (Leica).

### 2.4. RNA Isolation, cDNA Synthesis and Real-Time Quantitative PCR

ADMECs were seeded onto different matrices previously cut into small pieces with a biopsy punch under sterile conditions. Cells were cultured for 36 h at 37 °C in a humidified atmosphere in a 5% *v*/*v* CO_2_ incubator. RNA was extracted from cells with a Total RNA purification kit (Norgen Biotek Corp., Thorold, ON, Canada) following the manufacturer’s protocol, and retrotranscribed to cDNA with the SuperMix kit (Bioline, Meridian Life Science, Memphis, TN, USA) as previously described [28]. The expression level of the genes of interest was evaluated by comparative quantification based on the reaction efficacy and normalized to the expression of *18S*, *GAPDH*, and *TBP* as housekeeping genes [29]. The expression level of the following genes was investigated: *VEGFA, PGF, ANGPT1, KDR, FLT1, TEK, IL6, IL8/CXCL8, TNF, MCP1/CCL2, MMP2*, and *MMP9*. Primer sequences are reported in Table 2:

The reaction was performed using the Rotor-Gene 6000 (Corbett Research, Mortlake, Australia), using SYBR™ Green PCR Master Mix (Applied Biosystems, Milan, Italy), following a program of 45 cycles of denaturation (60 s at 95 °C), annealing (30 s at 60 °C, the melting temperature of the primers) and amplification (60 s at 72 °C).

### 2.5. MTT Assay

Matrices were cut into small pieces (8 mm in diameter) using a biopsy punch under sterile conditions. ADMECs were seeded onto the matrices at a concentration of 2.5 × 10^5^ cells/matrix. After 5, 15 or 30 min of adhesion at 37 °C in a 5% CO_2_ *v/v* incubator, nonadherent cells were washed away with dPBS supplemented with 0.7 mM CaCl_2_ and 0.7 mM MgCl_2_. To analyze cell viability, 3-(4,5-dimethylthiazol-2-yl)-2,5-diphenyltetrazolium bromide (MTT) was added and incubated at 37 °C for 2–4 h. The purple formazan crystals were solubilized by adding dimethyl sulfoxide (DMSO) under shaking for 20 min. Absorbance was read at 570 nm using a plate reading spectrophotometer.

### 2.6. Time Course Adhesion Assay

Matrices were cut into small pieces (8 mm in diameter) using a biopsy punch under sterile conditions. Cells were stained with 10 μg/mL of fluorescent dye FAST DiI (molecular probes, Invitrogen) diluted in dPBS. After 15 min of incubation at 37 °C in a 5% *v/v* CO_2_ incubator, 2.5 × 10^5^ cells were resuspended in HESFM supplemented with 10% FBS and 1:100 hydrocortisone and seeded onto the matrices. After 5, 15 or 30 min of adhesion at 37 °C in a 5% *v/v* CO_2_ incubator, nonadherent cells were washed away with dPBS supplemented with 0.7 mM CaCl_2_ and 0.7 mM MgCl_2_. Cells were lysed and the plate was immediately read with Infinite200 (TECAN Italia, Milan, Italy).

### 2.7. Proliferation Assay

ADMECs were cultured for 4 days in an incubator at 37 °C, 5% *v/v* CO_2_. After 4 days, matrices were labelled with 10 μg/mL of FAST DiI diluted in dPBS and lysing buffer was added. The fluorescence was read with Infinite200 (TECAN Italia, Milan, Italy).

### 2.8. Staining for Ki-67 and Vimentin

Matrixes were cut into small pieces (8 mm in diameter) with a biopsy punch under sterile conditions. ADMECs were seeded onto the matrices and cultured for 4 days in an incubator at 37 °C, 5% *v/v* CO_2_. Matrices were fixed with 3% PFA for 20 min at RT under shaking in the darkness. Matrices were incubated for 40 min at RT under shaking with primary antibodies anti-Ki-67 and antivimentin diluted in saponin A, composed of 60 mg Saponin (Farmitalia Carlo Erba, Milan, Italy) in 10 mL dPBS; and for 30 min at RT in darkness under shaking with LI-COR Biosciences-specific secondary antibodies. Images were acquired using Odissey CLx (LI-COR Biosciences, Lincoln, NE, USA) and analyzed with LI-COR Image Studio Acquisition software (LI-COR Biosciences, Lincoln, NE, USA).

To chemically mimic hypoxia and diabetes, cells were cultured for 4 days in the presence of 100 μM deferoxamine (Sigma Aldrich) or 20 μM advanced glycation endproduct-BSA (AGE-BSA), kindly donated by Dr. Cristina Bellarosa (Italian Liver Foundation). The concentration of deferoxamine was selected based on a previous work by Sánchez-Elsner et al. [30]. The concentration of AGE-BSA was determined by previous studies by Gallo et al. [31]. The seeding and staining protocol are described above.

### 2.9. Growth Factors and Chemokines Detection

ADMECs were seeded onto different matrices previously cut into small pieces with a biopsy punch under sterile conditions, or grown to confluence in 24-well plates (BD Falcon) in serum-free medium and stimulated for 24 h with interferon (IFN)-γ (100 U/mL), tumor necrosis factor (TNF)-α (100 ng/mL) or interleukin (IL)-1β (10 ng/mL) (all purchased from Peprotech, Milan, Italy). Cells seeded onto matrices were cultured for 36 h at 37 °C in a humidified atmosphere in a 5% *v*/*v* CO_2_ incubator; the supernatant was then collected. The levels of placental growth factor (PlGF), VEGF-A, TNF-α, and IL-8 were determined with a commercial ELISA kit following the manufacturer’s protocol (Human PlGF ELISA kit, Human ANGPT-1 ELISA kit and Human VEGF-A ELISA kit, Sigma Aldrich; Human TNF-α ELISA KIT, Diaclone SAS; IL-8 Human ELISA Kit, Invitrogen Thermo Fisher Scientific).

The initial quantitative determination of IL-8/CXCL8, monocyte chemoattractant protein (MCP)-1/CCL2, macrophage inflammatory protein (MIP)-1α/CCL3, regulated upon activation, normal T cell expressed and secreted (RANTES)/CCL5, and IL-6 was performed by a bead-based multiplex immunoassay (Biorad) and a Bioplex 200 system (Biorad Laboratories, Hercules, CA, USA), as previously described [32].

### 2.10. Subcutaneously Application of ADMECs and In Vivo Experiments

First, 2.5 × 10^5^ ECs were resuspended in HESFM and seeded for 30 min at 37 °C, 5% *v/v* CO_2_, Integra^®^ Dermal Regeneration Template without a silicone layer (Integra^®^ Life Science Corporation) or PELNAC^®^ Dermal Substitute (Eurosurgical), that had previously been cut into small pieces (6 mm × 5 mm) using a sterile scalpel. After cell seeding, the scaffolds were directly implanted on the quadriceps of immunodeficient NOD scid gamma (NSG) mice [14]. Surgical implantation of the scaffolds was performed on adult NSG (8 weeks old) mice under general anesthesia by ketamine/xylazine (100 mg/kg and 10 mg/kg body weight, i.p.). An incision of approximately 1 cm was made at the level of the knee towards the medial thigh on the left hind limb of the mice. The skin was separated from the quadricep muscle and the scaffolds were applied with the cells facing down. The incision was closed with 5–0 suture thread. Institutional guidelines in compliance with national and international laws and policies were followed for animal care and treatment. All experimental procedures were approved by the ICGEB Animal Welfare Board, with the requirements of the EU Directive 2010/63/EU, and by the Italian Ministry of Health.

The mice were sacrificed by cervical dislocation after anesthesia with 5% isoflurane. Integra^®^, PELNAC^®^ and the surrounding tissue on the quadricep were collected, transversely cut in half and fixed overnight using 2% *v/v* PFA (paraformaldehyde) in dPBS 1X solution (Santa Cruz). The following day, PFA solution was removed and replaced with 20% sucrose cryoprotector solution (Sigma-Aldrich, St. Louis, MO, USA). The samples were cut into 8-μm sections using cryostat. The sections were thawed and dried at room temperature for 5 min. After a wash in 1X dPBS, samples were permeabilized using 0.5% *v/v* Triton X-100 in 1X dPBS for 15 min. The blocking step was performed using 5% *w/v* in 1X dPBS solution for 1 h. The sections were incubated overnight at 4 °C using a humid chamber with the primary antibody, diluted at a ratio of 1:200 *v/v* with 1% *v/v* BSA, 0.1% *v/v* Tween-20 in 1X dPBS solution. The next day, the slides were washed once in 0.02% *v/v* Tween 20 in 1X dPBS solution for 5 min and twice in 1X dPBS for 5 min each. The incubation with secondary antibody was performed for 2 h at RT in a humid chamber diluting the secondary antibody 1:500 *v/v* with 1% *v/v* BSA, 0,1% *v/v* Tween-20 in 1X dPBS solution. The tissues were washed once in 0.02% *v/v* Tween 20 in 1X dPBS solution for 5 min and twice in 1X dPBS for 5 min each. Nuclei were counterstained with Hoechst 33342 (#H3570 Invitrogen) diluted 1:5000 in 1X dPBS for 7 min. Subsequently, slides were washed three times with 1X dPBS and mounted using Mowiol mounting medium (Sigma-Aldrich).

## 3. Results

### 3.1. Phenotypic Characterization of ADMECs and Evaluation of their Responsiveness to Pro-Inflammatory Cytokines

**Figure 1:**
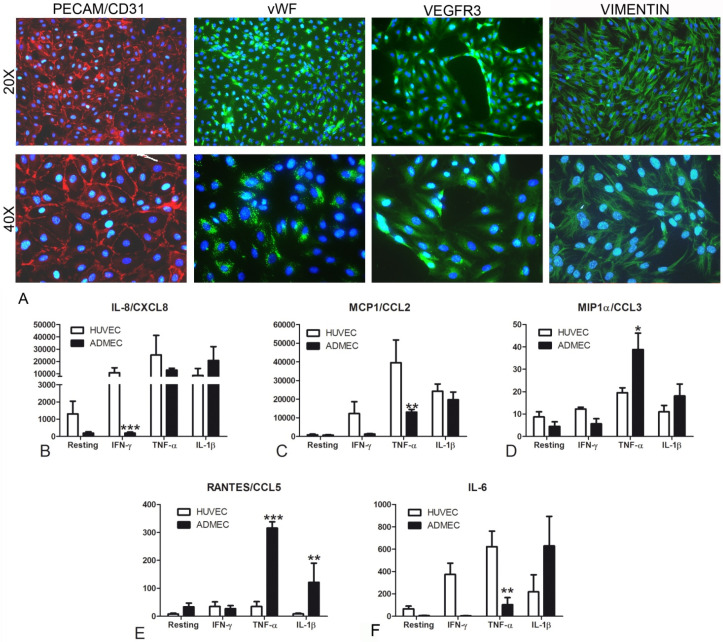
Phenotypic characterization of adult dermal microvascular endothelial cells (ADMECs). (**A**) Immunofluorescence (IF) analysis of PECAM/CD31 (in red), vWF, VEGFR3 and vimentin (in green) of isolated and cultured ADMECs. Scale bar 50 μm. The production of IL-8/CXCL8 (**B**), MCP1/CCL2 (**C**), MIP1α/CCL3 (**D**), RANTES/CCL5 (**E**) and IL-6 (**F**) was measured in the supernatant of HUVECs and ADMECs stimulated for 4 h with TNF-α using a bead-based multiplex immunoassay (Luminex^®^). The data represent the mean ± standard error (SE) of triplicate samples from five separate experiments * *p* < 0.05; ** *p* < 0.01; *** *p* < 0.005.

ADMECs were isolated from skin biopsies of adult patients undergoing reductive plastic surgery. We carried out an extensive characterization by immunofluorescence (IF) (Figure 1A) in order to determine the purity of the isolated cells. ADMECs were positively stained for classical vascular endothelium markers such as PECAM/CD31, vWF, VEGFR3 and Vimentin, but were completely negative for Podoplanin, CK8/18 and CD45 (data not shown). We evaluated the behavior of ADMECs in response to classical inflammatory stimuli, as compared to HUVECs (Figure 1B–F). As reported in Figure 1B, ADMECs manifested a low responsiveness to IFN-γ and TNF-α stimulation in terms of IL-8/CXCL8, MCP1/CCL2 and IL-6 secretion, but expressed higher levels of MIP1α/CCL3, RANTES/CCL5, TNF-α and IL-1β stimulation.

### 3.2. Evaluation of ADMEC Adhesive Properties and Colonization Capability of Different Dermal Substitutes

**Figure 2:**
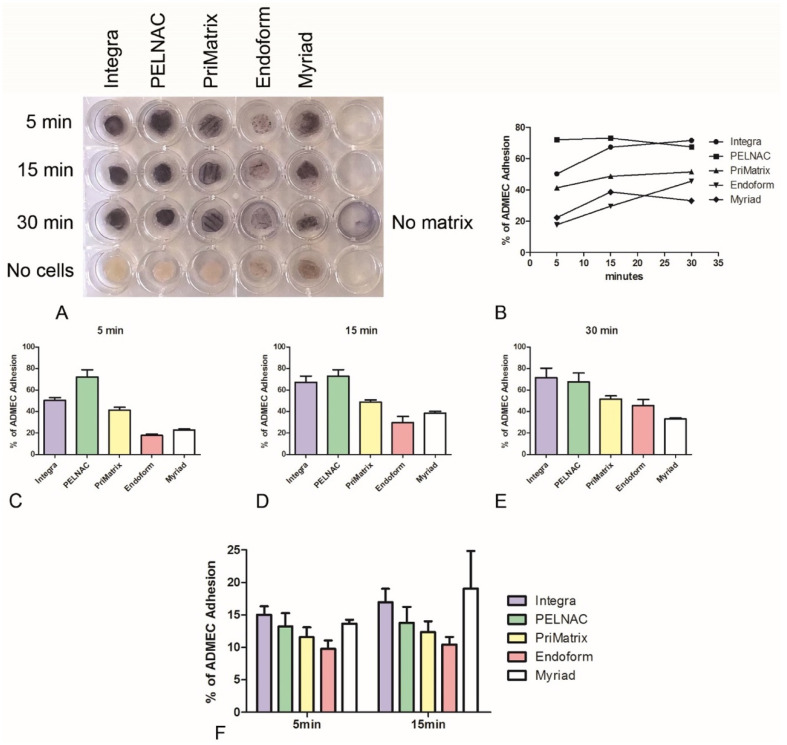
(**A**–**E**) ADMEC adhesion on Integra^®^, PELNAC^®^, PriMatrix^®^, Endoform^®^, or Myriad^®^ was evaluated by MTT assay. ADMECs were seeded onto the different matrices and incubated for three different durations (5, 15 and 30 min). After the removal of nonadherent cells, MTT was added and matrices were incubated at 37 °C for almost 4 h. (**A**) Picture of 24-well plate used for MTT assay before the solubilization of formazan crystals. (**B**–**E**) Formazan crystals were solubilized by adding DMSO; the absorbance was read at 570 nm by a plate reading spectrophotometer. Results are expressed as percentage of cell adhesion with reference to the absorbance obtained with the total number of seeded cells plated on fibronectin (the calculation formula is reported in the Supplementary Material). (**F**) ADMECs were labelled with the fluorescent dye FAST Dil, seeded onto different dermal substitutes and incubated for 5 or 15 min. After washing out the nonadherent cells from the scaffolds, labelled cells were lysed to release the dye in solution; the fluorescence was read with an Infinite200 TECAN reader. Results are expressed as percentage of cell adhesion with reference to a calibration curve established with an increasing number of labelled cells. Data from six independent experiments are presented as mean ± SE.

ADMECs were seeded onto five different dermal substitutes (Integra^®^, PELNAC^®^, PriMatrix^®^, Endoform^®^, or Myriad^®^). After 5, 15 or 30 min of incubation, the scaffolds were extensively washed to remove unattached cells, and adherent ADMECs were labelled with the viability marker MTT in a 24-well plate (Figure 2A). Successively, MTT crystals were solubilized with DMSO and absorbance was read at 570 nm. As shown in Figure 2B,C, Integra^®^ and PELNAC^®^ demonstrated superior pro-adhesive properties compared to PriMatrix^®^, Endoform^®^ and Myriad^®^. We observed that the cells were able to colonize the entire thickness/depth of these scaffolds (Videos S1 and S2), as demonstrated by IF analysis with antihuman CD31. The higher pro-adhesive properties of Integra^®^ and PELNAC^®^ were further confirmed by labelling ADMECs with FAST DiI, a viable fluorescent dye, prior to seeding. After 5 or 15 min of adhesion, labelled cells were lysed and the fluorescence was quantified. In this case, Myriad^®^ showed the greatest ability to stimulate cell adhesion, but also a high variability among the different ADMEC populations. The results confirmed data obtained by the MTT technique, highlighting the slightly better behavior of Integra^®^ in terms of EC colonization capability.

### 3.3. Assessment of the Proliferative Drive Induced by the Different Dermal Substitutes

**Figure 3:**
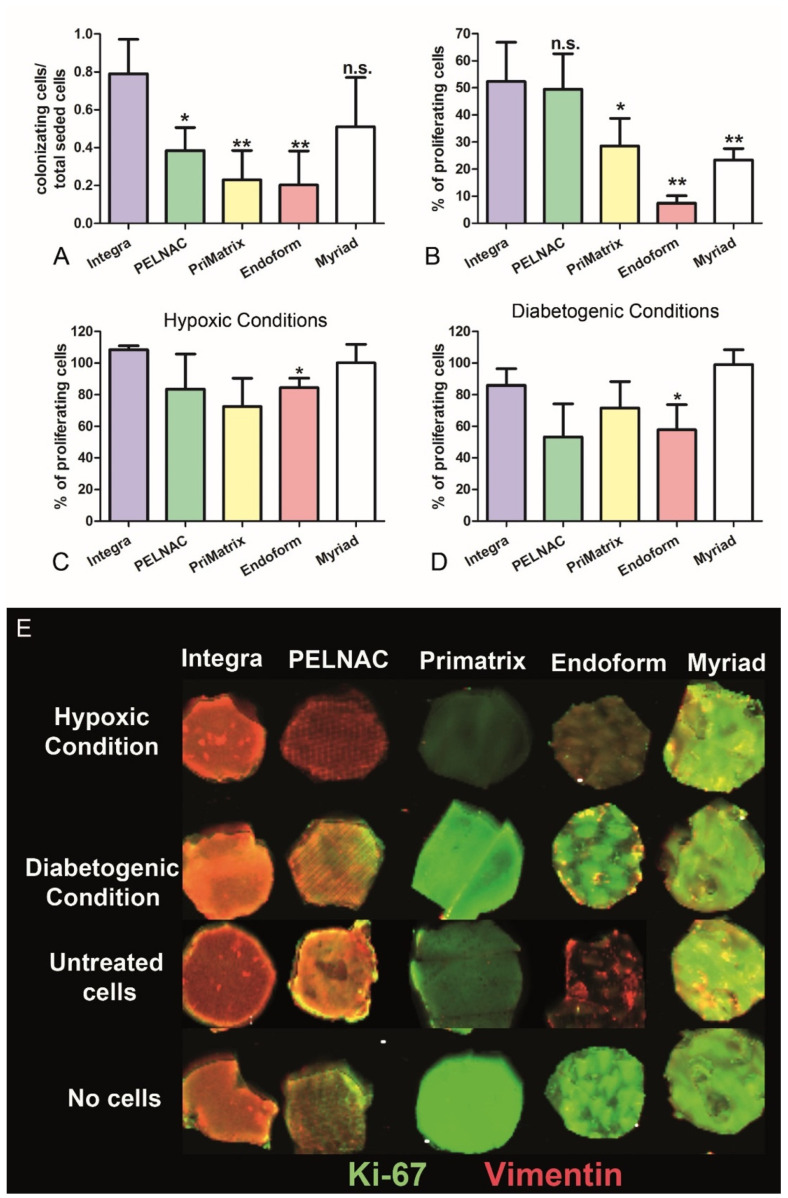
(**A**) After 36 h of culture onto the different dermal substitutes, ADMECs were stained with the fluorescent dye FAST Dil; labelled cells were lysed to release the dye in solution, and the fluorescence was read at 353 nm with Infinite200 TECAN reader. Results are expressed as a ratio between the fluorescence of the cells seeded in the scaffold and the fluorescence of the total number of cells (the calculation formula is reported in the Supplementary Material). Data from five independent experiments are presented as mean ± SE. * *p* < 0.05; ** *p* < 0.01 vs. Integra^®^. (**B**–**E**) The cell-colonized scaffolds under resting conditions (**B**), under hypoxic conditions (**C**) or diabetogenic conditions (**D**) were fixed and stained with mouse antihuman vimentin and rabbit antihuman Ki67 antibodies and analyzed by the fluorescent scanner Odyssey CL-x (LI-COR Biosciences, Lincoln, NE, USA). (**E**) A representative image of labelled scaffolds was acquired by using LI-COR Odyssey imaging system, and data were processed using Image Studio system 5.0 software (LI-COR Biosciences, Lincoln, NE, USA). The formula used for the calculation is reported in the Supplementary Material section. Data from four independent experiments conducted in triplicate are presented as mean ± SE. * *p* < 0.01 vs. Integra; n.s. = not significant.

In order to evaluate the proliferative boost induced by the different dermal substitutes, ADMECs were grown onto the scaffolds for 36 h and then labelled with FAST DiI. As shown in Figure 3A, the presence of living ADMECs was significantly higher on Integra^®^ compared to the other scaffolds, except Myriad^®^, which demonstrated highly variable behavior. To determine the percentage of proliferating cells present in the different dermal substitutes, the attached cells were stained for both vimentin in order to determine the total number of cells, and for Ki-67 to calculate the number of proliferating cells. Both stainings were quantified using a Biosciences Infrared Odyssey imaging system (LI-COR Biosciences, Lincoln, NE, USA), a fluorescence scanner which is able to detect multiple layers on the z-axis. As reported in Figure 3B, ADMECs grown on Integra^®^ and PELNAC^®^ exceeded a proliferation percentage of 40%, whereas those on PriMatrix^®^, Endoform^®^ and Myriad^®^ showed a lower proliferation index.

With the aim of mimicking a chronic wound microenvironment, we cultured ADMECs under hypoxic conditions (i.e., by incubating with deferoxamine) or diabetogenic conditions (i.e., by adding AGE-BSA to the culture media). The results shown in Figure 3C,D indicate that there were no significant differences among the proliferative rates of cells seeded onto the different scaffolds, except for Endoform^®^, which manifested a statistically lower proliferative rate in both conditions. Even if not significantly, Integra^®^ and Myriad^®^ demonstrated the best performance under hypoxic and diabetic conditions.

### 3.4. Proangiogenic and Remodeling Factors Produced by ADMECs on Different Dermal Substitutes

Having demonstrated the poor pro-adhesive and proproliferative properties of PriMatrix^®^, Endoform^®^, and considering the great variability of the results obtained with Myriad^®^, subsequent studies were conducted only on Integra^®^ and PELNAC^®^. To assess the mRNA expression of angiogenic-promoting factors (VEGFA, PGF, ANGPT1), receptors (KDR, FLT1, TEK) and tissue remodeling factors (MMP2 and MMP9) expressed by ADMECs cultured on Integra^®^ and PELNAC^®^, we performed RT-qPCR after seeding the cells onto the scaffolds for 36 h.

**Figure 4:**
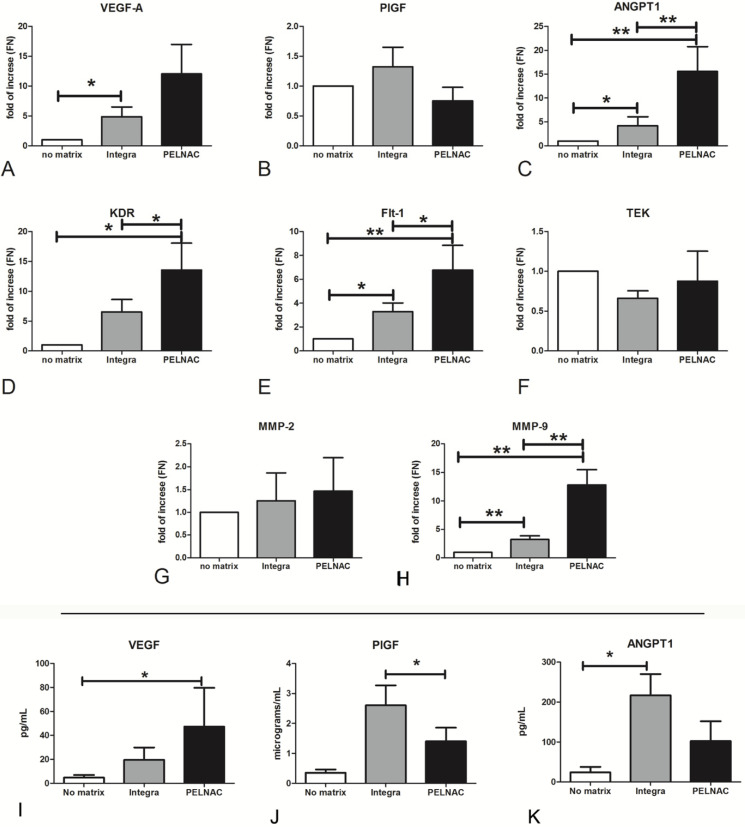
(**A**–**H**) RT-qPCR of VEGFA, PlGF, ANGPT1, KDR, FLT1, TEK, MMP2 and MMP9 expressed by ADMECs cultured onto different matrices for 36 h. The expression level of the genes is described as fold of increase with respect to the mean of normalized values of 18S, GAPDH and TBP as housekeeping genes. Data are expressed as the mean ± SE of duplicate samples from four separate experiments: * *p* < 0.05, ** *p* < 0.01. (**I**–**K**) Production of VEGF-A, PlGF and ANGPT-1 by ADMECs cultured on matrices. Thirty-six hours after ADMEC, the supernatant was collected and protein concentration was determined by ELISA. Data are presented as the mean ± SE of duplicate samples from three separate experiments.

The interaction of ADMECs with Integra^®^ induced the activation of almost all angiogenetic factor genes compared to 2D cell culture (except for TEK and MMP2). PELNAC^®^ was able to activate numerous angiogenic genes and, in some cases, in an even stronger manner than Integra^®^ (AGPT1, KDR, FLT1, MMP9) (Figure 4A–H).

Moreover, the upregulation of soluble angiogenetic factors (VEGF-A, PlGF and ANGPT-1) was also confirmed in terms of protein secretion (Figure 4I–K).

### 3.5. Dermal Substitutes Modulate the Pro-Inflammatory Behavior of ADMEC

**Figure 5:**
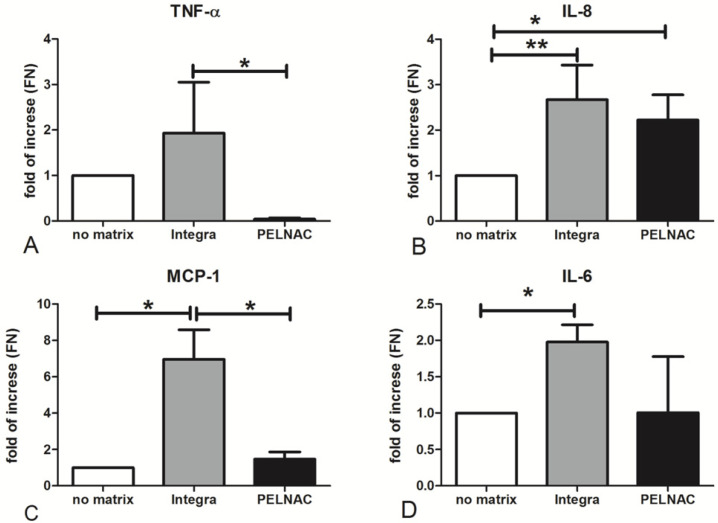
(**A**–**D**) RT-qPCR of TNF, IL8/CXCL8, MCP1/CCL2 and IL6 expressed by ADMECs cultured onto different matrices. Data represent the mean ± SE of duplicate samples from three separate experiments: * *p* < 0.05, ** *p* < 0.01.

Since the formation of granulation tissue and leukocyte recruitment is fundamental for the *restitutio ad integrum* of wounds [14], we wanted to investigate the ability of the different scaffolds to induce ADMEC expression of the chemokine and cytokine genes involved in the inflammatory process. The results shown in Figure 5A–D indicate that Integra^®^ was able to upregulate the expression of the healing-promoting cytokines as follows: a two-fold increase was observed in the expression of TNF, a 2.5-fold increase in the upregulation of IL8/CXCL8 was observed; a seven-fold increase of MCP1/CCL2 was observed; and a two-fold of IL6 was observed, as compared to cells seeded without scaffolds. On the other hand, PELNAC^®^ yielded only a two-fold upregulation of IL8.

The cytokine upregulation observed for gene expression was not confirmed at the protein level. We confirmed a slight, nonsignificant upregulation of IL-8/CXCL8 (Figure S1A), but we detected a significant downregulation of TNF-α (Figure S1B).

### 3.6. In Vivo Studies

**Figure 6:**
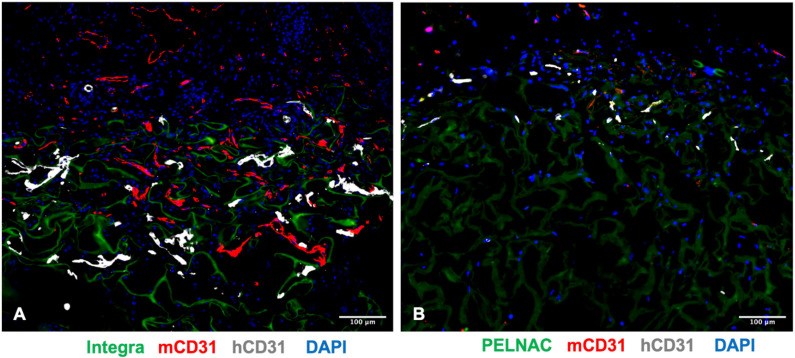
ADMECs engrafted and formed vascular structures on Integra^®^ but not on PELNAC^®^. Representative images of the Integra^®^ (**A**) and PELNAC^®^ (**B**) scaffolds, populated by ADMECs at day 10 after subcutaneous cell implantation. Human and mouse ECs were stained using species-specific anti-CD31 antibodies. Both Integra^®^ and PELNAC^®^ are visible in green due to scaffold autofluorescence. Scale bar 100 μm.

To determine the capacity of ADMECs to support the vascularization of clinically employed dermal regeneration scaffolds in vivo, we used host immunodeficient NSG mice to assess the engraftment and persistence of these cells following subcutaneous implantation. ADMECs were seeded either on Integra^®^ or PELNAC^®^ for 30 min and subcutaneously implanted on the quadriceps of NSG recipient mice. Histological analysis at 10 days revealed the presence of CD31-stained human ECs that colonized Integra^®^ (Figure 6A). In some cases, we observed the formation of human derived vascular structures inside the scaffold that were approaching mouse ECs, stained for mouse CD31. In addition, the host ECs colonized Integra^®^, demonstrating the successful engraftment of the scaffold beside the application and its vascularization. In contrast, very few CD31^+^ cells, of either human or mouse origin, were present inside PELNAC^®^, and they did not form any vascular structures (Figure 6B). Overall, these data indicate that Integra^®^ is a more suitable scaffold for tissue engineering approaches, as it promotes a high level of EC engraftment, integration and vascularization.

## 4. Discussion

Tissue-engineered skin substitutes are essential for tissue repair and regeneration when dealing with wounds which are large in size and penetrate deep below the dermis [33]. Although skin was the first engineered organ that went from laboratory research to patient care, there are still several limitations on the use of dermal substitutes, such as reduced vascularization, poor mechanical integrity, failure to integrate, scarring and immune rejection [34]. In the present study, we report on the differential ability of clinically employed dermal regeneration scaffolds to support ADMECs for the revascularization process in a chronic wound microenvironment.

We selected five different dermal substitutes which are currently being used in clinics (see Table 1), i.e., Integra^®^ Bilayer Matrix Wound Dressing, PELNAC^®^, PriMatrix^®^ Dermal Repair Scaffold, Endoform^®^ Natural Dermal Template, and Myriad Matrix^®^, and compared their capacity to be colonized by adult dermal microvascular ECs, inducing angiogenesis. Integra^®^ Bilayer Matrix Wound Dressing is a double layer membrane consisting of a porous coprecipitate of type I bovine cross-linked tendon collagen, rich in shark chondroitin-6-sulfate and a removable silicon layer. PELNAC^®^ is a dermal regenerating matrix composed of atelocollagen derived from pig tendon and reinforced with a silicone layer. PriMatrix^®^ Dermal Repair Scaffold is derived from fetal bovine dermis and is particularly rich in type III collagen that is active in developing and healing tissues. Endoform^®^ Natural Dermal Template is a recently developed matrix based on AORA ECM^®^ technology. It is derived from decellularized ovine forestomach, harvested exclusively from New Zealand pasture-raised animals. This bioscaffold contains more than 150 ECM proteins which are important for the wound healing process, and maintains residual vascular channels to support the establishment of new vasculature. Myriad Matrix^®^ is an engineered ECM that contains the natural porous structure of AROA ECM^®^ with interstitial perforations that allow cell infiltration.

Microvascular ECs are active participants in, and regulators of, inflammatory processes, secreting inflammatory mediators, modulating adhesion and migration of leukocytes through the expression of adhesion molecules and chemokines [28]. As demonstrated by Agostinis et al. [28], ECs are not all the same, and they respond very differently depending on the tissue of origin. In order to demonstrate the differential behavior of ECs of different provenance in response to inflammatory conditions, we compared ADMECs isolated from adult dermal biopsies with HUVECs isolated from human umbilical veins, which have been extensively characterized in terms of angiogenesis and inflammation. ADMECs were found to be less responsive to IFN-γ compared to HUVECs, although they expressed a high level of cytokines and chemokines in response to TNF-α and IL-1β. Their low responsiveness to pro-inflammatory cytokines, such as IFN-γ, resulted in a lower production of IL-8/CXCL8, MCP1/CCL2 and IL-6. In contrast, stimulation with TNF-α and IL-1β increased the production of MIP1α/CCL3 and RANTES/CCL5, two chemokines involved in lymphocyte and neutrophil recruitment and secreted at very high levels by ADMECs. Based on these differences, the use of ADMECs rather than HUVECs appeared to be more promising for our in vitro model. Moreover, we decided to use ADMECs for an easier translational feasibility, since the future application of autologous ADMECs, isolated from patient punch biopsies, would be desirable to improve wound healing in clinical practice.

The principal aim of this study was to enhance revascularization to successfully promote wound healing [35]. Since the first step of matrix colonization is cell adhesion, we performed adhesion assays to assess the different adhesive properties of the five scaffolds. All of the analyzed matrices, with the exception of the Endoform^®^, reached a plateau value after just 15 min. Integra^®^ and PELNAC^®^ showed excellent pro-adhesive properties after 5 min, making them the most promising scaffolds. Myriad^®^ also showed some interesting adhesive features despite its variability among replicates and cell populations. Another property under evaluation was the abilities of the scaffolds to induce EC proliferation. To this end, we used two different approaches: (i) quantification of the total number of living cells present in the matrix after 36 h, and (ii) the percentage of proliferating cells (positive for Ki-67) normalized for the total number of cells (positive for vimentin). The results confirmed those obtained for the adhesion experiments, indicating that Integra^®^, PELNAC^®^, and Myriad^®^ were the most promising dermal substitutes in terms of local proliferation of vascular cells.

Chronic wounds are defined as a discontinuity in the skin barrier lasting longer than 42 days. Risk factors for the delay of physiological wound healing are chronic disease, diabetes, age, vascular insufficiency, nutritional deficit and some local factors [36]. With the aim of mimicking a chronic wound microenvironment, we cultured ADMECs in the scaffolds under hypoxic conditions by using deferoxamine. Furthermore, we wanted to reproduce diabetogenic conditions by adding AGE-BSA to the culture media, as advanced glycation end products and their receptors (RAGE) have been shown to be involved in the pathogenesis of diabetes [37]. The results suggested that only cells seeded onto Myriad^®^ were able to proliferate under the hypoxia condition. In general, hypoxia signaling is essential during tissue and organ development, but this condition is also important in specific organs in healthy adults. In fact, since the hypoxia-inducible factor(HIF)-α is not hydroxylated by prolyl hydroxylase under hypoxic conditions, it can activate the HIF-mediated transcriptional program, which also includes angiogenesis. Hypoxia triggers the transcription of angiogenic genes including *VEGFA, PGF, PDGFB* and *ANGPT1* and *2*. Furthermore, it posttranscriptionally controls pro-angiogenic chemokines and receptors, inducing the migration of progenitors to the site of angiogenesis. Hypoxia can also promote EC proliferation and sprouting in addition to matrix remodeling [38]. The physiological wound healing process requires both angiogenesis, which means the sprouting of newly formed capillaries from pre-existing blood vessels, and vasculogenesis, which implies the formation of new vessels by recruited progenitor cells. Wound healing failure is linked with aging and diabetes, as well as their synergistic negative effects. Several studies have demonstrated that HIF-α expression is decreased in a diabetic mouse model, and that its inactivity in mice exposed to high glucose concentrations could be reversed by treatment with deferoxamine, increasing wound vascularization and healing in *db/db* mice [39].

Nonenzymatic glycation of intra- and extra- cellular proteins by hyperglycemia may cause tissue damage. In fact, AGEs irreversibly bind to proteins. Several studies have shown that AGEs induce endothelial dysfunction. In particular, they increase oxidant stress, induce permeability, stimulate the expression of adhesion molecules and augment the migration of T cells and macrophages [40]. For this reason, we decided to culture cells in the presence of AGE-BSA to mimic diabetogenic conditions. The results of our experiments revealed that all three dermal substitutes (Integra^®^, PELNAC^®^ and Myriad^®^) were able to support at least 10% of proliferating cells in a diabetes-like condition.

We successively focused our experiments on Integra^®^ and PELNAC^®^ because they demonstrated the highest vascularization properties and low variability in the results. The angiogenic drive induced by dermal substitutes was evaluated by RT-qPCR for the main pro-angiogenic factors/receptors and tissue remodeling factors. With the exception of *TEK*, the ANGPT-1 receptor, both matrices (Integra^®^ and PELNAC^®^) were shown to activate the expression of all genes under study. Furthermore, we also confirmed, at the protein level, the upregulation of VEGF-A synthesis by PELNAC^®^ of and the upregulation of PlGF secretion by Integra^®^.

Inflammation is one of the physiological responses to wound healing [13]. The formation of granulation tissue is a *condition sine qua non* to complete *restitutio ad integrum*. Thus, we performed RT-qPCR with the aim of evaluating the expression of healing-promoting cytokines. In this case, Integra^®^ was shown to be able to upregulate the gene expression of *TNF*, *IL8/CXCL8*, *MCP1/CCL2* and *IL6*. These data were partially confirmed at the protein level, since Integra^®^ seemed to downregulate the production of TNF-α, but also to increase the production of IL-8/CXCL8. In this case, Integra^®^ also showed the most promising results by upregulating the genes involved in leukocyte recruitment, e.g., IL-8/CXCL8 and MCP1/CCL2, although this also led to the upregulation of TNF-α and IL-6, cytokines involved in chronic inflammation.

Finally, in vivo experiments were undertaken to confirm our in vitro observations. We assessed the ability of preseeded ADMECs on Integra^®^ to promote the engraftment, integration and vascularization of clinically employed dermal regeneration scaffolds at the site of application. In vivo experiments to evaluate the efficacy of human cell therapies require the use of immunocompromised mice (in our case NSG mice) [14]. Given that they allow the engraftment of human cells, these mice are widely used in preclinical assessments of human cell-based therapies despite the absence of the complex immune response required for wound healing [41].

## 5. Conclusions

In conclusion, Integra^®^, PELNAC^®^ and Myriad^®^ demonstrated the best performance in terms of EC colonization and proliferation, even under hypoxic and diabetic conditions. The interaction of ECs with both Integra^®^ and PELNAC^®^ induced the expression of multiple angiogenetic factors. Integra^®^ was the most effective scaffold in promoting EC engraftment and vascularization in vivo. Thus, the implantation of dermal ECs in combination with ECMs represents a promising strategy for tissue revascularization. Integra^®^ remains the “gold standard” ADM for this application.

## Figures and Tables

**Table 1:** Summary of the characteristics of the dermal substitutes analyzed in the current study. Dermal substitute structures were designed using the Blender 3D software (Blender Foundation, Stichting Blender Foundation, Buiklotermeerplein, Amsterdam, The Netherlands).

	Commercial Scaffolds	Components
** 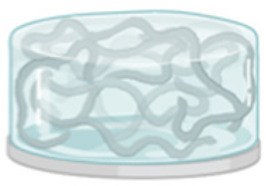 **	Integra^®^ Bilayer Matrix Wound Dressing	The dermal layer is composed of bovine type I tendon collagen and chondroitin-6-sulfate; the epidermal layer is made of silicon.
** 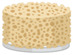 **	PELNAC^®^	A bilayer scaffold made of porcine tendon atelocollagen and a silicon layer.
** 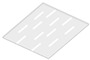 **	PriMatrix^®^ Dermal Repair Scaffold	Acellular dermal tissue matrix from fetal bovine dermis, particularly rich in type II collagen.
** 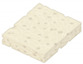 **	Endoform^®^ Natural Dermal Template	Derived from ovine forestomach tissue that is minimally processed to separate tissue layers and decellularize the tissue extracellular matrix (ECM). It maintains components essential to tissue repair (e.g., collagen I, III, IV, fibronectin, laminin, elastin, hyaluronic acid, heparin sulfate, GAGs, growth factors and chemokines).
** 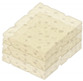 **	Myriad Matrix^®^	A collagen matrix with an intact ECM. Derived from ovine forestomach, it retains the innate biological structure and function of the native ECM associated macromolecules including elastin, fibronectin, glycosaminoglycans and laminin.

**Table 2:** Primer sequences used for the Real-Time qPCR analysis.

	Gene	Tm	Sense	Sequence (5′ → 3′)	Accession Number
Ribosomal protein S18	*RPS18*	60	Forward Reverse	ATC CCT GAA AAG TTC CAG CA CCC TGT TGG TGA GGT CAA TG	NM_022551.2
Glyceraldehyde-3-phosphate dehydrogenase	*GAPDH*	60	Forward Reverse	GAT CAT CAG CAA TGC CTC CT GT GGT CAT GAG TCC TTC CA	NM_002046.5
TATA-box binding protein	*TBP*	60	Forward Reverse	GAG CCA AGA GTG AAG AAC AGT C GCT CCC CAC CAT ATT CTG AAT CT	NM_003194.4
Vascular endothelial growth factor A	*VEGFA*	60	Forward Reverse	CCT GGT GGA CAT CTT CCA GGA GT CTC ACC GCC TCG GCT TGT CAC A	NM_001025366.2
Placental growth factor	*PGF*	62	Forward Reverse	GAA CGG CTC GTC AGA GGT G ACA GTG CAG ATT CTC ATC GCC	NM_001207012
Angiopoietin 1	*ANGPT1*	60	Forward Reverse	AGC GCC GAA GTC CAG AAA AC TAC TCT CAC GAC AGT TGC CAT	NM_001146
Kinase insert domain receptor	*KDR*	60	Forward Reverse	GGC CCA ATA ATC AGA GTG GCA CCA GTG TCA TTT CCG ATC ACT TT	NM_002253
Fms related tyrosine kinase 1	*FLT1*	62	Forward Reverse	GAA AAC GCA TAA TCT GGG ACA GT GCG TGG TGT GCT TAT TTG GA	NM_001159920
TEK receptor tyrosine kinase	*TEK*	62	Forward Reverse	CAG GAT ACG AAC CAT GAA GAT GC GGG GCA CTG AAT GGA TGA AG	NM_000459
Interleukin 6	*IL6*	60	Forward Reverse	GTA CAT CCT CGA CGG CAT C CCA GGC AAG TCT CCT CAT TG	NM_000600.3
C-X-C motif chemokine ligand 8	*IL8/CXCL8*	60	Forward Reverse	AGG TGC AGT AGT TTT GCC AAG GA TTT CTG TGT TGG CGC AGT GT	NM_000584
Tumor necrosis factor	*TNF*	65	Forward Reverse	GGC CCA GGC AGT CAG ATC AT GGG GCT CTT GAT GGC AGA GA	NM_000594.3
C-C motif chemokine ligand 2	*MCP1/CCL2*	60	Forward Reverse	ATC AAT GCC CCA GTC ACC AGT CTT CGG AGT TTG GG	NM_002982.3
Matrix metallopeptidase 2	*MMP2*	60	Forward Reverse	TAC AGG ATC ATT GGC TAC ACA CC GGT CAC ATC GCT CCA GAC T	NM_004530
Matrix metallopeptidase 9	*MMP9*	60	Forward Reverse	GGG ACG CAG ACA TCG TCA TC TCG TCA TCG TCG AAA TGG GC	NM_004994

## Data Availability

The data presented in this study are available within the article and in the Supplementary Materials.

## Supplementary Materials

The following supplementary materials are available online at https://www.mdpi.com/article/10.3390/biomedicines9101458/s1: Figure S1: Secretion of TNF-α and IL-8/CXCL8 by ADMECs cultured on matrices; Supplementary Information: Calculation formulas; Video S1: 3D immunofluorescence of endothelial cell-colonized Integra^®^; Video S2: 3D immunofluorescence of endothelial cell-colonized PELNAC^®^.
