# Supplementary material for: Differential Capability of Clinically Employed Dermal Regeneration Scaffolds to Support Vascularization for Tissue Bioengineering
Source: Biomedicines. 2021 Oct 13;9(10):1458. doi: 10.3390/biomedicines9101458 (PMC8533449; doi:10.3390/biomedicines9101458)
Supplement: Supplementary file 1 [file biomedicines-09-01458-s001.zip › Supplemental Material.pdf]

Supplementary Material to

Article

Differential capability of clinically employed dermal regeneration scaffolds to support vascularization for tissue bioengineering

Chiara Agostinis [1], Mariagiulia Spazzapan [2], Roman Vuerich [2,3], Andrea Balduit [2,*], Chiara Stocco [4], Alessandro Mangogna [1], Giuseppe Ricci [1,4], Giovanni Papa [4], Serena Zacchigna [3,4], and Roberta Bulla [2]

1. Institute for Maternal and Child Health, I.R.C.C.S. "Burlo Garofolo", 34137, Trieste, Italy; chiara.agostinis@burlo.trieste.it (C.A.); alessandro.mangogna@burlo.trieste.it (A.M.); giuseppe.ricci@burlo.trieste.it (G.R.)
2. Department of Life Sciences, University of Trieste, 34127, Trieste, Italy; mariagiulia.spazzapan@studenti.units.it (M.S.); roman.vuerich@icgeb.org (R.V.); rbulla@units.it (R.B.)
3. Cardiovascular Biology Laboratory, International Centre for Genetic Engineering and Biotechnology, 34149, Trieste, Italy; serena.zacchigna@icgeb.org (S.Z.)
4. Department of Medical, Surgical and Health Sciences, University of Trieste, 34100, Trieste, Italy; chiarastoccomd@gmail.com (C.S.); giovanni.papa@asugi.sanita.fvg.it (G.P.)

* Correspondence: abalduit@units.it (A.B.); Tel.: +390405588653; Department of Life Sciences, University of Trieste, via Valerio 28, 34127, Trieste, Italy.

Supplementary Figures

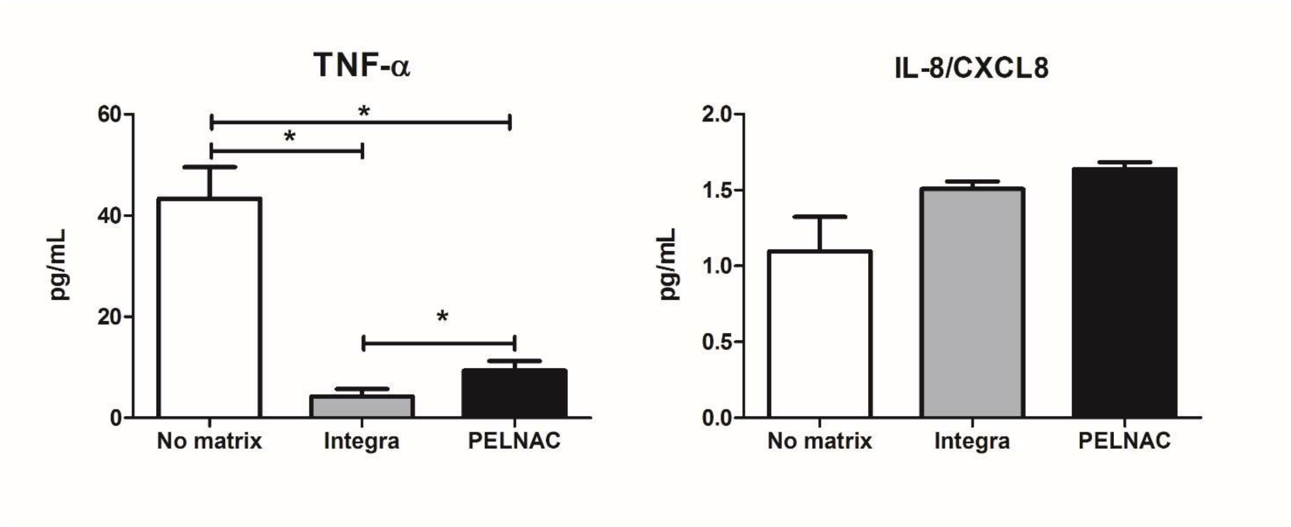

Figure S1. Secretion of TNF-α and IL-8/CXCL8 by ADMECs cultured on matrices. After 36 h from ADMEC seeding, supernatant was collected and protein concentrations were determined by ELISA following the manufacturer's protocol. Data represent the mean ± SE of duplicate samples from three separate experiments.

Supplementary Information: Calculation Formulas

Formula Figure 2B-E

$$\frac{Sample\ value\ (O.D.570nm) - No\ Cells}{No\ matrix} * 100 = \%\ of\ ADMEC\ adhesion$$

Formula Figure 2F

$$\frac{Sample\ value\ (F.U.) - No\ cells}{No\ Matrix} * 100\ = \%\ of\ ADMEC\ adhesion$$

Formula Figure 3A

$$\frac{Sample\ value\ (F.U.)}{No\ matrix\ Total\ cells\ (F.U.)} = colonizating\ cells\ /\ total\ seeded\ cells$$

Formula Figure 3B

$$\frac{Sample\ value\ Ki67 - No\ cells}{Sample\ value\ VIM - No\ cells} * 100\ = \%\ of\ proliferating\ cells$$

Formula Figure 3C,D

$$\frac{Sample\ value\ Ki67}{Sample\ value\ Ki67\ resting\ condition} * 100\ = \%\ of\ proliferating\ cells$$

Supplementary Videos

Video S1. 3D immunofluorescence of endothelial cell-colonized Integra®.

Video S2. 3D immunofluorescence of endothelial cell-colonized PELNAC®.
